# Clinical efficacy of Jianqiao ancient moxibustion in treating chronic fatigue syndrome: a single-center randomized controlled superiority trial protocol

**DOI:** 10.3389/fmed.2026.1860390

**Published:** 2026-06-26

**Authors:** Qimiao Hu, Bonuo Qi, Yingjun Liu, Haijuan Zhang, Yulin Liu, Yuping Yang, Chao Wang

**Affiliations:** 1 The Third Clinical Medical College, Zhejiang Chinese Medical University, Hangzhou, China; 2 The Third Affiliated Hospital of Zhejiang Chinese Medical University (Zhongshan Hospital of Zhejiang Province), Hangzhou, China; 3 Pan’an Hospital of Traditional Chinese Medicine, Jinhua, China; 4 Hangzhou Lin’an Traditional Chinese Medicine Hospital, Hangzhou, China

**Keywords:** ancient moxibustion, chronic fatigue syndrome, infrared thermal imaging, protocol, randomized controlled superiority trial

## Abstract

**Background:**

Chronic fatigue syndrome (CFS) is a widespread and debilitating disorder that profoundly impacts the physical and mental health of patients. In traditional Chinese medicine (TCM), spleen–kidney yang deficiency is recognized as the most prevalent syndrome associated with CFS. Moxibustion is commonly employed to mitigate fatigue and address yang deficiency; however, conventional moxa box moxibustion has demonstrated limited clinical efficacy in practice. Considering the intrinsic benefits of Jianqiao ancient moxibustion in terms of its herbal formula and treatment coverage, this randomized controlled superiority trial aims to compare the clinical efficacy of Jianqiao ancient moxibustion with that of moxa box moxibustion in patients with CFS who exhibit a spleen–kidney yang deficiency pattern.

**Methods:**

This single-center, single-blind randomized controlled superiority trial will enroll 90 eligible patients diagnosed with CFS exhibiting a spleen–kidney yang deficiency pattern. Participants will be randomly assigned in a 1:1 ratio to receive either Jianqiao ancient moxibustion or moxa box moxibustion, with each group receiving standardized interventions as outlined in the trial protocol. Each 60-min treatment session will be administered three times per week for 4 consecutive weeks. A follow-up assessment will be conducted at week 16. The primary outcome measure will be the Fatigue Scale-14. Secondary outcome measures will include the Spleen–Kidney Yang Deficiency Symptom Scale, the Self-Rating Anxiety Scale, the Self-Rating Depression Scale, and infrared thermal imaging. All evaluations will be performed at baseline, immediately post-intervention at week 4, and during follow-up at week 16.

**Discussion:**

It is hypothesized that Jianqiao ancient moxibustion will be more effective than moxa box moxibustion in alleviating symptoms in patients with CFS who have spleen–kidney yang deficiency. Furthermore, this study will employ infrared thermal imaging to assess alterations in the thermal characteristics of the Sanjiao and Governor Vessel both prior to and following moxibustion. This methodology may help elucidate the mechanisms underlying moxibustion’s modulation of yang-qi distribution in patients with CFS.

**Study protocol registration:**

https://www.chictr.org.cn/showproj.html?proj=193045.

## 1 Introduction

Chronic fatigue syndrome (CFS) is a common idiopathic disorder characterized by unexplained fatigue that persists for at least 6 months. In addition, it is accompanied by at least four secondary symptoms, including unrefreshing sleep, prolonged post-exertional malaise, impairment of concentration or short-term memory, sore throat, tender lymph nodes, multi-joint and muscle pain, and headaches (1). The exact pathogenesis of CFS has yet to be fully elucidated. Nevertheless, existing evidence suggests the potential implication of infectious agents, psychological stressors, metabolic disturbances, and genetic predispositions. Furthermore, functional abnormalities in the immune, neural, endocrine, and intestinal microbiota systems are implicated in the pathophysiology of CFS (2, 3). Globally, an estimated 1.7 to 24 million individuals are affected by CFS, with approximately 836,000 to 2.5 million of these individuals residing in the United States. Epidemiological data indicate that the estimated prevalence of CFS is 0.89% according to the Fukuda criteria, with female participants affected approximately 1.5 times more frequently than male individuals (4, 5). In China, the estimated prevalence of CFS in the general population is 12.54%, with a similarly high prevalence observed among female individuals (6). Clinically, between 10 and 25% of patients experience impaired mobility, 25.7% lose their working capacity, and 16.2% are severely confined to bed, requiring long-term care (7). Due to its debilitating physical and cognitive symptoms, which are exacerbated by activity, CFS also imposes significant socioeconomic burden (8). At present, while the symptoms of CFS can be managed, there remains no definitive cure for the condition, whether through pharmacological or non-pharmacological means. According to the 2021 NICE guidelines, essential management strategies encompass energy management, the integration of physical activity and exercise, symptomatic treatment, and multidisciplinary care. Importantly, in contrast to the 2007 NICE guidelines, the updated 2021 guidelines advise against using graded exercise therapy for CFS and clarify that cognitive behavioral therapy is not curative (9, 10). Consequently, there continues to be a pressing clinical demand for the development of innovative and efficacious therapies for CFS.

Traditional chinese medicine (TCM) does not have a distinct term for CFS; instead, it is categorized under consumptive disease based on clinical manifestations (11). The central pathogenesis of this condition is identified as yang-qi deficiency (12). Importantly, within TCM, the kidney is considered the congenital foundation and the spleen is considered the acquired foundation of the human body. Consequently, a deficiency in spleen–kidney yang-qi can result in impaired visceral function (13). Furthermore, spleen–kidney yang deficiency syndrome is the most prevalent pattern observed in the clinical syndrome differentiation of CFS (14). As a traditional non-pharmacological therapy within TCM, moxibustion has historically been considered a primary intervention for consumptive diseases (15). This technique involves the combustion of moxa wool to provide thermal stimulation to the body surface. It regulates the balance of qi and blood by warming the meridians, unblocking collaterals, and enhancing qi and blood circulation, ultimately restoring the body’s yang-qi vitality (16). Numerous studies have demonstrated its significant efficacy in patients with CFS, showing substantial alleviation of fatigue, depressive mood, sleep disturbances, and other related symptoms (17).

Conventional moxa box moxibustion demonstrates partial efficacy in the treatment of CFS; however, it is limited by restricted treatment coverage, suboptimal thermal penetration, and incomplete symptomatic improvement. Jianqiao ancient moxibustion is a distinctive form of moxa stick moxibustion recognized as a representative element of traditional medicine and as an intangible cultural heritage of Hangzhou, Zhejiang Province (18). This practice employs a proprietary formula that integrates southern herbs, known as “Jian Shiba,” with northern moxa, as detailed in Table 1. Furthermore, it incorporates specialized traditional processing techniques, including herbal decoction, natural sun-drying, and manual pounding into moxa floss (19). The resulting product comprises two large moxa sticks, noted for their significant thermal intensity and enhanced penetrative capacity. Owing to its extensive contact area and unique herbal composition, Jianqiao ancient moxibustion is theoretically posited to exert superior effects in regulating yin-yang, nourishing qi and blood, and unblocking meridians. Considering the theoretical advantages of Jianqiao ancient moxibustion and the inherent limitations of moxa box moxibustion, this study proposes a randomized controlled superiority trial to compare their clinical efficacy in patients with CFS exhibiting a spleen–kidney yang deficiency pattern.

**Table 1:** Authentic Chinese medicinal materials of “Southern herbs and Northern moxa”.

Category	Place of origin	Composition
“Southern herbs” (“Jian Shiba”)	Jianqiao Area, Shangcheng District, Hangzhou	Scrophularia ningpoensis (Radix Scrophulariae) *Ophiopogon japonicus* (Radix Ophiopogonis) Rehmannia glutinosa (Radix Rehmanniae) Mentha haplocalyx (Herba Menthae) *Cassia obtusifolia* (Semen Cassiae) *Euphorbia lathyris* (Semen Euphorbiae) Angelica dahurica (Radix Angelicae Dahuricae) *Brassica alba* (Semen Sinapis) Schizonepeta tenuifolia (Herba Schizonepetae) *Arctium lappa* (Fructus Arctii) *Lycopus lucidus* (Herba Lycopi) *Ricinus communis* (Semen Ricini) *Benincasa hispida* (Semen Benincasae) *Benincasa hispida* (Exocarpium Benincasae) *Raphanus sativus* (Semen Raphani) *Raphanus sativus* (Dried Radish Root) Eupolyphaga sinensis (Eupolyphaga) *Bombyx mori* (Bombyx Batryticatus)
“Northern moxa”	Nanyang, Henan	Moxa (Artemisia argyi)

## 2 Methods

### 2.1 Trial design

This study is a single-center, single-blind, randomized controlled superiority trial. A total of 90 eligible participants diagnosed with CFS with a spleen–kidney yang deficiency pattern will be enrolled. Participants will be randomly assigned in a 1:1 ratio to receive either Jianqiao ancient moxibustion or moxa box moxibustion. The study will span 16 weeks, comprising a 4-week treatment period and a subsequent 12-week follow-up phase.

**Figure 1:**
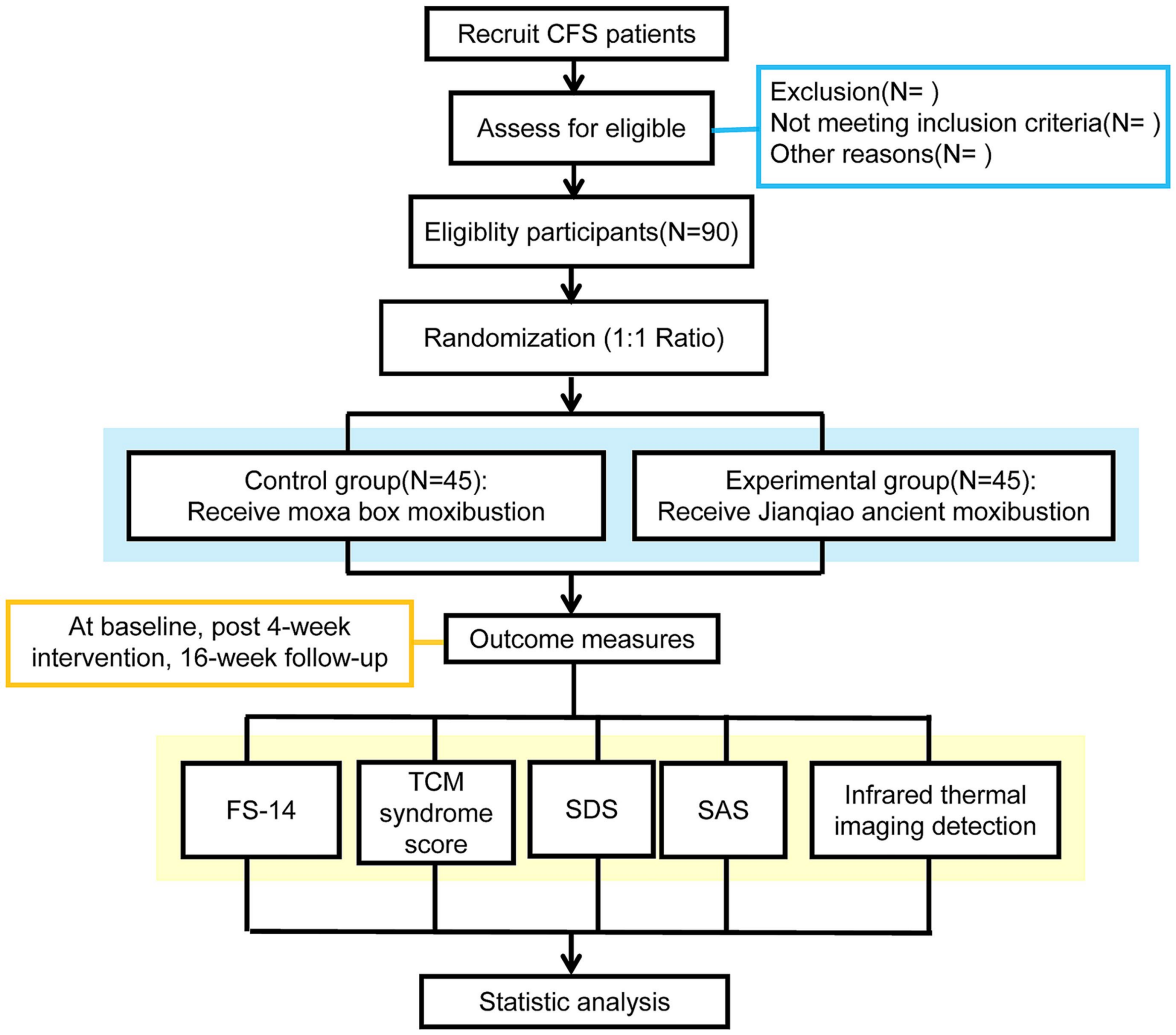
Flowchart of the trial.

The trial is registered with the Chinese Clinical Trial Registry (ChiCTR2300079165). The trial flowchart is illustrated in Figure 1, and a detailed timeline is provided in Table 2.

**Table 2:** Trial schedule.

Time point	Study period			
	Pre-treatment (Baseline) Week 0	Treatment Weeks 1–4	Post-treatment week 4	Follow-up week 16
Assessment of eligible				
Inclusion criteria	√	—	—	—
Exclusion criteria	√	—	—	—
Randomization				
Allocation	√	—	—	—
Intervention				
Jianqiao ancient moxibustion treatment	—	√	—	—
Moxa box moxibustion treatment	—	√	—	—
Primary outcome				
Fatigue Scale-14 (FS-14)	√	—	√	√
Secondary outcomes				
The Symptoms scale of Spleen–Kidney Yang Deficiency	√	—	√	√
Self-rating depression scale (SDS)	√	—	√	√
Self-rating anxiety scale (SAS)	√	—	√	√
Infrared thermal imaging detection	√	—	√	√

### 2.2 Study setting

This trial will be conducted at the Department of Acupuncture and Moxibustion at The Third Affiliated Hospital of Zhejiang Chinese Medical University. The department maintains a robust clinical patient volume, ensuring adequate participant recruitment. All study participants will be recruited from the inpatient and outpatient populations of this department. This prospective clinical trial is scheduled to take place from 1 January 2024 to 30 November 2026.

### 2.3 Research hypothesis

Primary hypothesis: Participants receiving Jianqiao ancient moxibustion will demonstrate significantly greater improvement in fatigue severity than those in the moxa box moxibustion group.

Secondary hypothesis: Participants in the Jianqiao ancient moxibustion group will exhibit significantly greater improvements in TCM syndrome scores and reductions in anxiety and depression levels. In addition, favorable alterations in the infrared thermal characteristics of the Sanjiao and Governor Vessel are expected.

### 2.4 Study participants

#### 2.4.1 Diagnostic criteria

1. Western Medical Diagnostic Criteria. The diagnostic criteria for CFS are formulated according to the 1994 Centers for Disease Control and Prevention (CDC) guidelines (9):
  (1) Patients suffer from unexplained persistent or recurrent chronic fatigue, regardless of physical strain, which cannot be alleviated by rest. The fatigue has a definite onset, results in substantial impairment of occupational, educational, or personal activities, and persists for six months or longer.
  (2) At least four of the following symptoms occur persistently or recurrently after fatigue onset: ① impaired memory or concentration that interferes with work, study, or social activities; ② sore throat; ③ tender palpable cervical or axillary lymph nodes; ④ myalgia; ⑤ multiple joint pain without redness or swelling; ⑥ newly developed severe headache; ⑦ unrefreshing sleep; and ⑧ fatigue that cannot be relieved within 24 h after exercise.
2. TCM Spleen–kidney Yang Deficiency Syndrome Diagnostic Criteria. These criteria are formulated according to the *Clinic Terminology of Traditional Chinese Medical Diagnosis and Treatment—Part 2: Syndromes/Patterns* (20):
  (1) Presence of at least three of the following five primary symptoms: ① fear of cold and cold extremities; ② lassitude and lack of strength; ③ shortness of breath and laziness to speak; ④ reduced food intake; and ⑤ soreness and weakness of the waist and knees.
  (2) Presence of at least two of the following six secondary symptoms: ① cold pain in the lumbar region; ② abdominal fullness and distention; ③ cold loose stools; ④ nocturia; ⑤ tooth-marked tongue; and ⑥ a deep, feeble pulse.

#### 2.4.2 Inclusion criteria

The inclusion criteria for this study are as follows:

1. Meeting the Western medical diagnostic criteria for CFS.
2. Meeting the TCM diagnostic criteria for consumptive disease exhibiting a spleen–kidney yang deficiency pattern.
3. Being aged 18–70 years, irrespective of gender.
4. Providing voluntary, written informed consent prior to participation.
5. Being free of severe organic diseases affecting major physiological systems.
6. Not receiving medication or participating in other clinical trials during the study period.

#### 2.4.3 Exclusion criteria

The exclusion criteria for this study are as follows:

1. Failure to meet the aforementioned Western medical or TCM diagnostic criteria.
2. Presence of primary organic, endocrine, neurological, or psychiatric diseases that could otherwise account for chronic fatigue.
3. History or presence of severe visceral, hematological, or immune system diseases, or malignant tumors.
4. Current use of other related treatments or concurrent enrollment in other clinical trials.
5. Women who are pregnant, lactating, or planning to conceive during the trial period.
6. Presence of cognitive impairment, severe mental disorders, alcohol or drug abuse, or anticipated poor compliance with the study protocol.
7. Presence of skin lesions, infection, or allergies at the targeted moxibustion sites, or scar constitution incompatible with moxibustion therapy.

#### 2.4.4 Elimination criteria

1. Mis-enrolled participants who do not meet the inclusion criteria.
2. Participants with incomplete data or those who commit serious protocol violations that compromise the efficacy assessment.
3. Participants demonstrating poor compliance, those who voluntarily withdraw, or those who utilize unauthorized alternative or prohibited treatments during the trial.
4. Trial termination due to severe adverse reactions or complications.

### 2.5 Randomization

This study will employ simple randomization to group participants. A third-party statistician will utilize SPSS version 22.0 to generate the randomization sequence, ensuring that researchers remain blinded to it. The random numbers are securely sealed in sequentially numbered opaque envelopes and stored in a double-locked cabinet. Upon completion of the baseline assessment and receipt of written informed consent, the envelopes will be opened sequentially in the order of enrollment to reveal group allocation. The allocation sequence will be maintained in strict confidentiality to prevent prediction of subsequent participants’ assignments.

### 2.6 Blinding

Due to the obvious differences in moxibustion devices and procedures, blinding of participants and practitioners is infeasible. To mitigate bias associated with awareness of treatment allocation, outcome evaluators and data statisticians will remain blinded throughout the trial. In addition to patient-reported outcomes, infrared thermal imaging will be utilized as an objective secondary outcome measure to minimize subjective assessment bias. Unblinding will be permitted solely in the event of severe adverse events or upon the completion of data analysis.

### 2.7 Interventions

**Figure 2:**
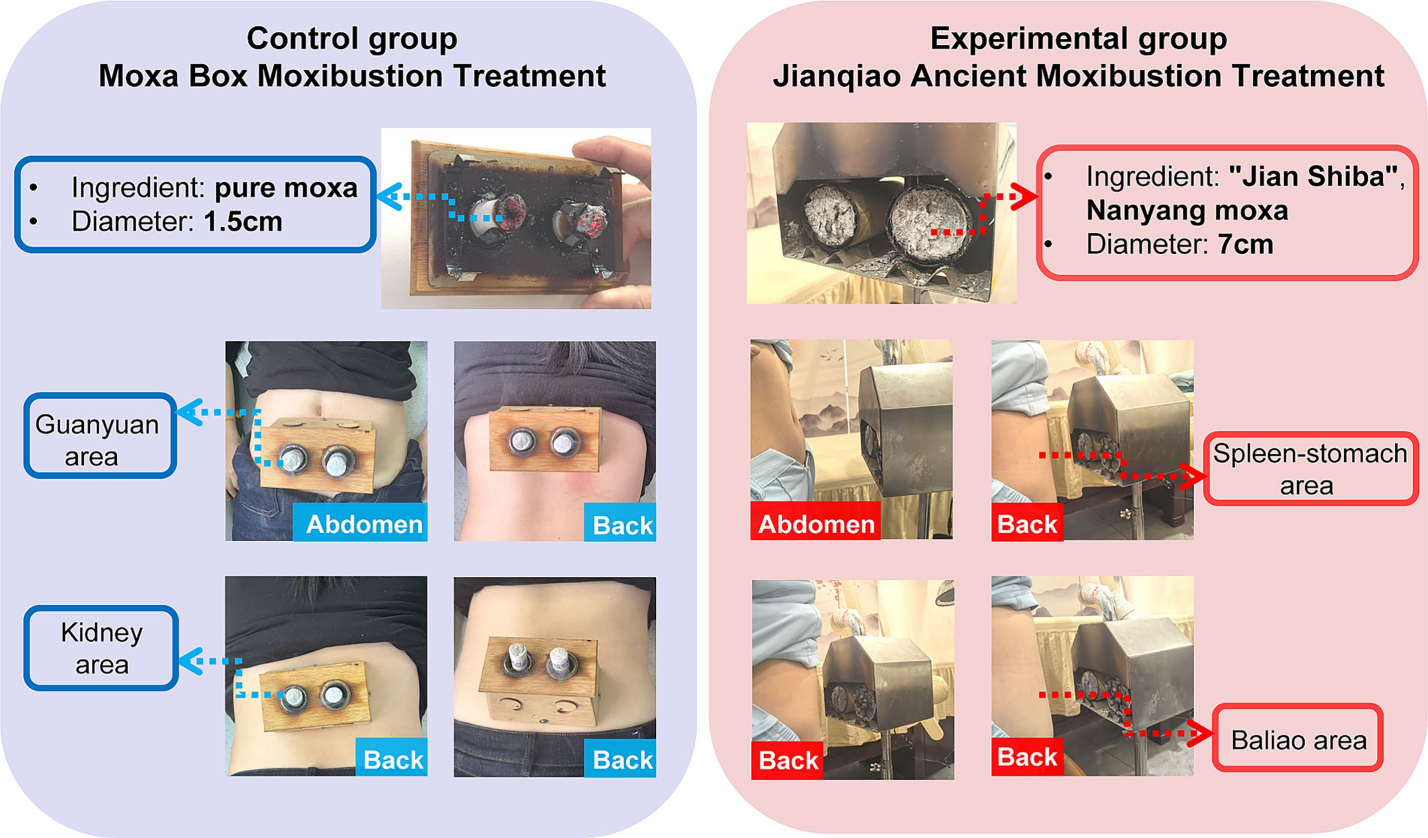
Comparison of intervention protocols between the control group and the experimental group.

A comparison of the intervention protocols between the moxa box moxibustion group and the Jianqiao ancient moxibustion group is illustrated in Figure 2.

#### 2.7.1 Experimental group: Jianqiao ancient moxibustion treatment

**Figure 3:**
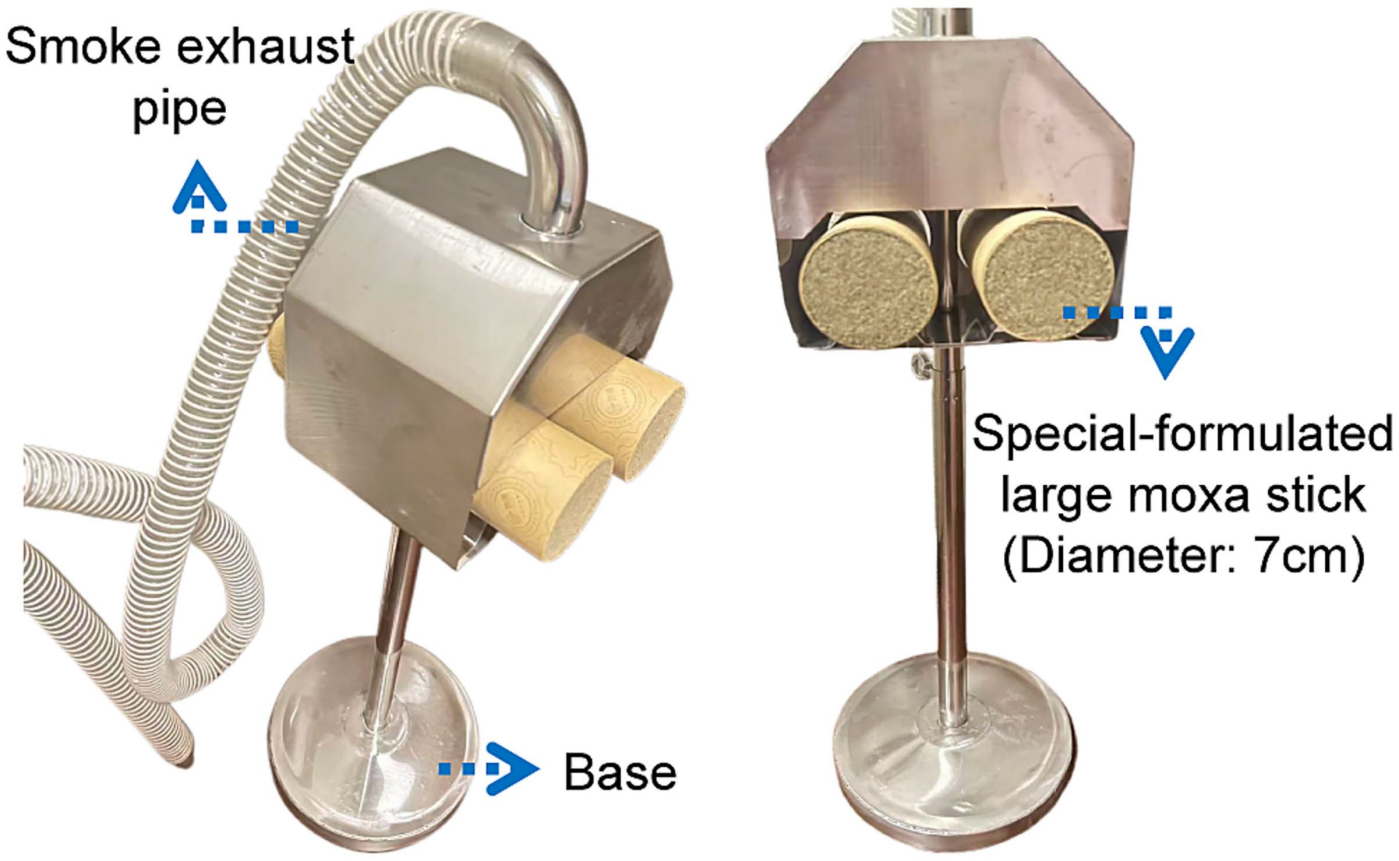
Special moxibustion device for Jianqiao ancient moxibustion.

**Figure 4:**
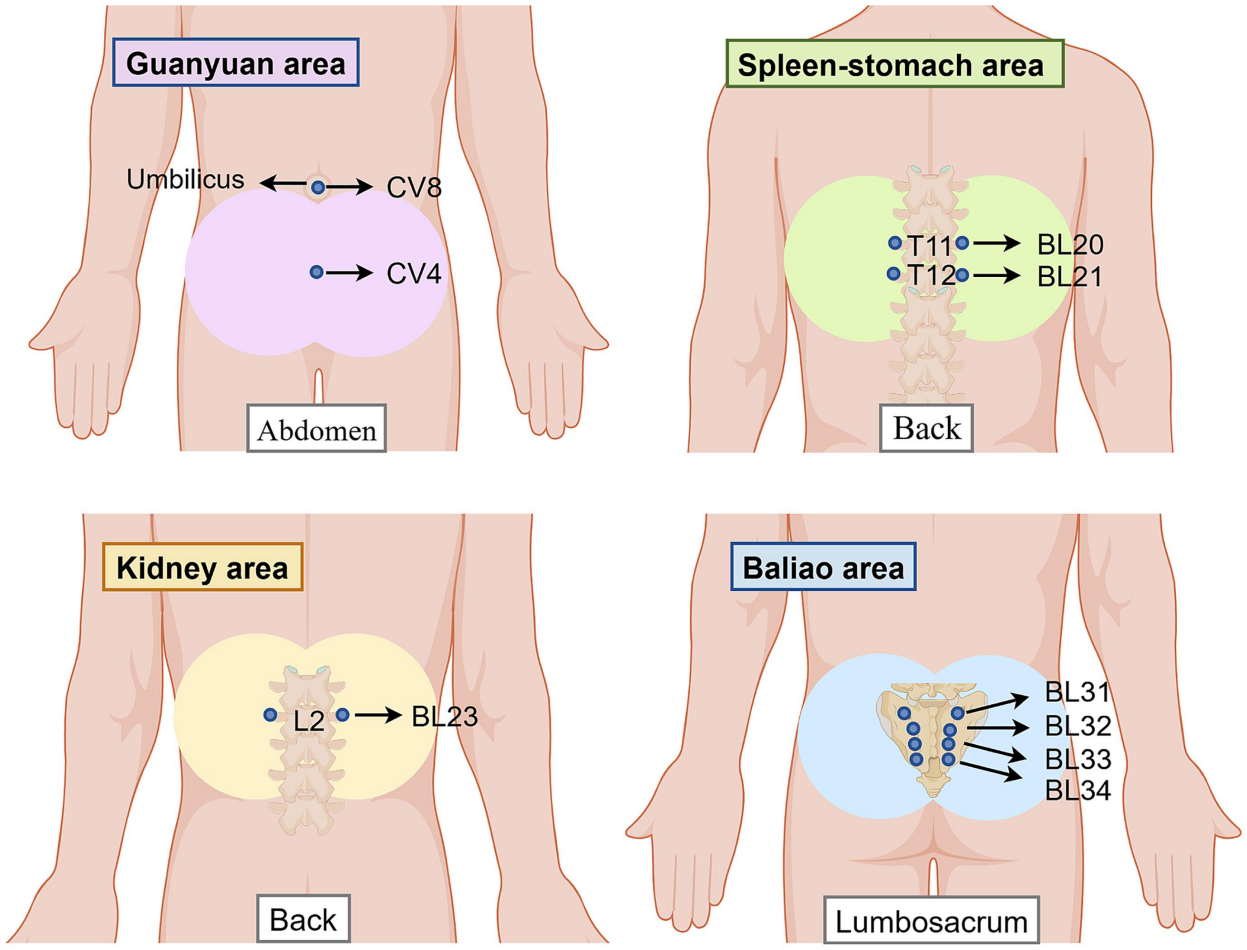
Schematic diagram of the Jianqiao ancient moxibustion areas.

1. Materials and equipment: Jianqiao ancient moxibustion specially formulated large moxa sticks (23 cm in length, 7 cm in diameter, and 500 g in weight), lighters, 75% alcohol cotton balls, and a specialized Jianqiao ancient moxibustion special moxibustion device. This device comprises a stainless steel three-hole box. Two horizontal rear holes are designed for moxa stick placement, and a vertical top hole connects to an exhaust fan and an external pipe to discharge smoke. The device is mounted on a height-adjustable stainless-steel bracket (Figure 3).
2. Administering practitioner: Licensed acupuncturists with a minimum of 3 years of clinical experience will conduct Jianqiao ancient moxibustion. Prior to study initiation, all practitioners will undergo standardized training and successfully pass an assessment. To ensure intervention consistency, a unified protocol will be rigorously enforced.
3. Preoperative precautions: The ambient temperature of the treatment room will be maintained at 26 °C. Moxibustion treatment will be postponed for patients who are fasting, have recently overeaten, or are experiencing acute exhaustion until they have sufficiently recovered.
4. Operating procedures: ① Position selection: Patients will initially assume a sitting posture with their backs facing the device to expose the lumbodorsal treatment areas. After lumbar and dorsal moxibustion, patients will reposition themselves to face the device, exposing the abdominal treatment areas. ② Moxibustion area selection: The targeted areas include the Guanyuan area (bilateral 7-cm-diameter circular areas on the abdomen covering CV4), the spleen–stomach area (bilateral 7-cm-diameter circular areas on the back covering ipsilateral BL20 and BL21), the kidney area (bilateral 7-cm-diameter circular areas on the lumbodorsum covering ipsilateral BL23), and the baliao area (bilateral 7-cm-diameter circular areas at the lumbosacrum covering ipsilateral BL31, BL32, BL33, and BL34). The schematic diagram of the moxibustion areas is shown in Figure 4. ③ Disinfection: The treatment areas will be routinely exposed and disinfected three times with 75% alcohol cotton balls. ④ Moxa stick ignition: The specially formulated large moxa sticks will be ignited and inserted into the two horizontal holes of the device. The exhaust fan will be activated, and the device will be positioned 15 cm away from the patient’s body surface. ⑤ Moxibustion operation: Suspended moxibustion will be performed sequentially on the kidney area, spleen–stomach area, and baliao area. Each dorsal site will receive 15 min of moxibustion until the local skin becomes flushed. Patients will then turn to face the device and receive another 15 min of moxibustion on the Guanyuan area until local skin flushing occurs. ⑥ Postoperative handling: The device will be removed after moxibustion is completed. Patients will be advised to keep warm and rest adequately.
5. Treatment frequency: Each of the four targeted acupoint areas will undergo moxibustion for 15 min, culminating in a total session duration of 60 min. The treatment protocol consists of three sessions per week for 4 consecutive weeks, resulting in a total of 12 sessions.

#### 2.7.2 Control group: moxa box moxibustion treatment

1. Materials and equipment: Standard pure moxa sticks (10 cm in length, 1.5 cm in diameter, and 20 g in weight), lighters, 75% alcohol cotton balls, and wooden dual-hole moxa boxes.
2. Administering practitioner: Licensed acupuncturists with a minimum of three years of clinical experience will administer the moxa box moxibustion. Prior to study initiation, all practitioners will undergo standardized training and successfully pass a competency assessment. To ensure intervention consistency, a unified protocol will be rigorously enforced.
3. Preoperative precautions: The ambient temperature will be maintained at 26 °C. Moxibustion should be postponed for patients who are fasting, have overeaten, or are experiencing severe fatigue until they have sufficiently recovered physically.
4. Operating procedures: ① Position selection: Patients will adopt a prone posture to expose the lumbodorsal treatment areas. After back moxibustion, they will switch to a supine position to expose the abdominal treatment areas. ② Moxibustion area selection: The Guanyuan area (bilateral 1.5-cm-diameter circular areas on the abdomen covering CV4), the spleen–stomach area (bilateral 1.5-cm-diameter circular areas on the back covering ipsilateral BL20 and BL21), the kidney area (bilateral 1.5-cm-diameter circular areas on the lumbodorsum covering ipsilateral BL23), the baliao area (two 1.5-cm-diameter circular areas per side at the lumbosacrum covering ipsilateral BL31, BL32, BL33 and BL34). ③ Disinfection: The treatment areas will be exposed and disinfected three times with 75% alcohol cotton balls. ④ Moxa stick ignition: Two moxa sticks will be ignited and inserted into the box holes, after which the box will be placed right above the targeted acupoints. ⑤ Moxibustion operation: Moxibustion will be applied sequentially to the kidney area, the spleen–stomach area, and the baliao area in a prone posture. Each area will be treated for 15 min until the local skin reddens and a mild warm sensation is felt. Patients will then turn supine and receive another 15-min moxibustion on the Guanyuan area following the same standard. Participants’ reactions will be closely monitored, and gauze will be placed under the box to relieve excessive heat stimulation if needed. ⑥ Postoperative handling: Following the session, the moxa box will be removed upon completion of treatment. Remaining moxa sticks and ashes will be properly disposed of, and patients will be advised to keep warm and rest adequately.
5. Treatment frequency: Each of the four targeted acupoint areas will undergo moxibustion for 15 min, culminating in a total session duration of 60 min. The treatment protocol consists of three sessions per week for 4 consecutive weeks, resulting in a total of 12 sessions.

### 2.8 Outcome measures

The assessment of outcomes encompasses the following domains: fatigue severity, TCM syndrome manifestations, symptoms of depression and anxiety, and body surface thermal characteristics. Assessments will be performed at baseline, immediately post-intervention (week 4), and during follow-up (week 16). Notably, infrared thermal imaging will be employed as an objective quantitative indicator, independent of subjective psychological perception, to evaluate body surface temperature distribution and thermal radiation characteristics along the meridians in each group.

#### 2.8.1 Primary outcome

The primary outcome measure will be the Fatigue Scale-14 (FS-14). The FS-14 is a validated 14-item questionnaire for evaluating physical and mental fatigue. Each item utilizes a dichotomous yes-or-no response format and is scored as 0 or 1, with a total score ranging from 0 to 14. Higher scores will indicate more severe chronic fatigue (21, 22). FS-14 assessments will be performed at baseline, week 4 (post-intervention), and week 16 (follow-up).

#### 2.8.2 Secondary outcomes

##### 2.8.2.1 The Spleen–Kidney Yang Deficiency Symptom Scale

The Spleen–Kidney Yang Deficiency Symptom Scale is a 4-grade TCM syndrome rating scale. It evaluates both primary and secondary symptoms. Primary symptoms include aversion to cold and cold extremities, lassitude and lack of strength, shortness of breath and reluctance to speak, reduced food intake, and soreness and weakness of the lower back and knees; each item is scored from 0 to 6. Secondary symptoms include lumbar cold pain, abdominal fullness and distention, loose stools, cold, increased urine volume at night, teeth-marked tongue, and a deep, weak pulse, each scored 0–3. Higher total scores denote more severe Spleen–Kidney Yang Deficiency (23, 24). The Spleen–Kidney Yang Deficiency Symptom Scale will be administered at baseline, week 4 (post-intervention), and week 16 (follow-up).

##### 2.8.2.2 Self-rating depression scale (SDS)

The SDS is a 20-item scale for assessing depressive symptoms, with total scores ranging from 20 to 80. Higher scores indicate greater depressive severity (25). The SDS will be administered at baseline, week 4 (post-intervention), and week 16 (follow-up).

##### 2.8.2.3 Self-rating anxiety scale (SAS)

The SAS is a 20-item scale used to assess anxiety symptoms, with total scores ranging from 20 to 80. Higher scores indicate greater anxiety severity (26). The SAS will be administered at baseline, week 4 (post-intervention), and week 16 (follow-up).

##### 2.8.2.4 Infrared thermal imaging detection

The distribution of body surface temperature is intricately linked to the functioning of visceral organs and meridians. Infrared thermal imaging, a non-invasive functional imaging modality, captures infrared radiation emitted by body surface tissues to depict superficial thermal characteristics (27). Infrared thermal imaging detection will be performed at baseline, week 4 (post-intervention), and week 16 (follow-up).

1. Instrument: The TTM-9000 medical infrared thermal imager (Hangzhou Xinhan Optoelectronics, Hangzhou, China) will be used. Its working waveband is 8–14 μm, the instantaneous field of view is ≤1.39 mrad, the temperature resolution is ≤0.1 °C, the measurement accuracy is ≤0.4 °C, and the detectable temperature range is 30–42 °C.
2. Pre-scan preparation: ① The ambient temperature will be set at 26 °C with a relative humidity of ≤ 70%. The scanning room will be kept free of obvious airflow and strong infrared radiation sources. ② Participants will be instructed to refrain from strenuous exercise, irritant foods, and medications for 24 h before scanning and to refrain from eating and bathing within 1 h prior. Female participants will avoid menstruation. ③ Participants will be asked to empty their bowels and bladder and remove all clothing, glasses, and accessories. Females will have their hair tied up to expose their necks. The skin will be allowed to dry naturally for 10 min before image collection.
3. Scanning protocol: ① Participant information will be registered in the supporting software. ② Participants will stand at the designated site for standard anterior and posterior trunk imaging. ③ The operator will adjust the camera parameters to acquire clear standard infrared thermal images. ④ Qualified images will be selected. The rectangular tool will be applied to delineate the Sanjiao and Governor Vessel regions. The division and zang-fu organs of the Sanjiao are presented in Table 3 (28). The mean temperature of each region will be recorded in an Excel spreadsheet. ⑤ All imaging data will be saved after confirmation.
4. Data processing: Alterations in surface thermal distribution and the Governor Vessel radiation trajectory will be compared before and after the intervention. The mean thermal values and their differences will be calculated. Quantitative analysis will be performed using the corresponding software platform for subsequent statistical analysis.

**Table 3:** Division and zang-fu organs of Sanjiao.

Sanjiao subdivision	Anatomical scope	Zang-fu organs
Upper jiao	Above the diaphragm	Heart and lung
Middle jiao	From the diaphragm to the umbilicus level	Spleen, stomach, liver, and gallbladder
Lower jiao	Below the umbilicus	Kidney, large intestine, small intestine, and bladder

### 2.9 Safety evaluation and adverse events

Continuous safety monitoring will be conducted throughout the intervention and follow-up periods. All adverse events, including but not limited to skin burns, blisters, local pruritus, cutaneous infections, dizziness, and allergic reactions, will be systematically observed and documented at each treatment and assessment visit. These events will be categorized into three severity levels: mild, moderate, and severe. Mild adverse events primarily consist of minor local itching and mild erythema that do not require clinical intervention. Moderate events are characterized by noticeable blisters, local infections, persistent dizziness, or allergic symptoms that necessitate symptomatic management. Severe events include significant skin burns, syncope, and other life-threatening conditions that require immediate medical intervention. Comprehensive details, including the onset time, clinical features, severity classification, and management strategies, will be meticulously recorded in the case report forms. The causal relationship between adverse events and moxibustion will be rigorously assessed. Qualified professionals will provide timely symptomatic treatment when adverse events occur. All severe adverse events will be promptly reported to the principal investigator and the institutional review board (IRB).

### 2.10 Data management and quality control

Participant data will be systematically recorded using standardized case report forms. To maintain confidentiality, participants will be anonymized with unique numerical codes, and all research data will be securely stored in an encrypted electronic database. The institutional ethics committee will provide continuous oversight of protocol implementation, the protection of participant rights, and the reporting of adverse events throughout the trial. Researchers involved in moxibustion procedures, scale evaluations, and infrared thermal imaging detection will undergo comprehensive standardized training prior to their participation in the study. Rigorous quality control measures will be applied to participant enrollment, randomization, intervention implementation, and outcome assessment, in strict adherence to the trial protocol. To minimize assessment bias, outcome assessors and statisticians will remain blinded to group allocations. Regular follow-up reminders will be scheduled to enhance participant compliance, and any instances of dropout, loss to follow-up, or protocol violations will be thoroughly documented. Regular verification of source data will be conducted, and updates on trial progress and adverse events will be promptly communicated to the ethics committee.

### 2.11 Sample size calculation

Sample size was determined by a previously published randomized controlled trial examining the effects of Governor moxibustion on patients with spleen–kidney yang deficiency pattern CFS, utilizing the FS-14 as the primary outcome measure (29). In that study, the post-treatment FS-14 scores were reported as 4.86 ± 1.43 for the moxibustion group and 6.23 ± 2.73 for the control group. The resulting Cohen’s d was 0.63, signifying a moderate effect size. To achieve a two-tailed significance level (*α*) of 0.05 and a statistical power of 0.80, the required sample size is determined to be 40 participants per group. Accounting for an anticipated 10% dropout rate, a total of 45 participants will be recruited per group, resulting in an overall sample size of 90 patients.

### 2.12 Statistical analysis

To mitigate subjective bias, all statistical analyses will be conducted independently by two blinded statisticians utilizing SPSS version 22.0. These statisticians will remain uninformed of group allocations and intervention specifics. The intention-to-treat (ITT) analysis set will include all randomized participants who complete the baseline assessment and receive at least one intervention session, and this set will be used for the primary efficacy analysis. The per-protocol (PP) analysis set will include subjects with good treatment adherence, no significant protocol deviations, and complete outcome data and will be utilized for sensitivity analysis to assess the robustness of the research findings. Data collected at multiple time points will be analyzed using a mixed-effects model for repeated measures, including group, time, and their interaction effects, to evaluate inter-group and temporal differences. Missing data will primarily be addressed through multiple imputation, supplemented by the last observation carried forward method for sensitivity analysis, to minimize bias resulting from data attrition. Covariates such as age, sex, disease duration, and baseline FS-14 scores will be adjusted for in the model to correct for confounding bias due to baseline imbalances. The Bonferroni correction will be employed to adjust for multiple comparisons, thereby controlling for the inflation of Type I error rates associated with simultaneous evaluations of multiple secondary outcomes. Treatment adherence will be quantified as the ratio of sessions actually completed to the total number of sessions planned; statistical comparisons of adherence between groups will be conducted, supplemented by additional sensitivity analyses stratified by adherence status. Continuous data exhibiting normal distribution will be analyzed using the *t*-test, whereas data not conforming to a normal distribution will be assessed via non-parametric rank-sum tests. Categorical variables will be compared using the chi-square test or Fisher’s exact test, as appropriate. All statistical tests will be two-tailed, with a significance threshold set at *a p*-value of < 0.05.

## 3 Discussion

CFS has become a significant global public health concern, characterized by an increasing incidence that profoundly affects patients’ physical and mental well-being, as well as their overall quality of life (30, 31). While moxibustion has been shown to effectively mitigate various symptoms associated with CFS, traditional moxa box moxibustion may have a limited ability to improve overall clinical outcomes. In pursuit of a more efficacious therapeutic approach, we have designed this trial.

Jianqiao ancient moxibustion and moxa box moxibustion share similarities in their operational methodologies, as both techniques utilize two moxa sticks to deliver thermal stimulation to the circular periacupoint area. However, they differ in moxa composition and stick size, which can lead to variations in their stimulatory effects. Specifically, moxa box moxibustion employs pure moxa, whereas Jianqiao ancient moxibustion incorporates a compound of southern herbs (“Jian Shiba”) and northern moxa. This formulation is purported to warm yang without depleting body fluids, clear heat without causing cold stagnation, and eliminate wind-phlegm without inducing dryness, thereby enhancing its efficacy in warming and tonifying yang-qi (19). Furthermore, regarding the stick diameter, moxa box moxibustion uses 1.5-cm sticks, whereas Jianqiao ancient moxibustion uses 7-cm sticks. The larger size of the moxa sticks in Jianqiao ancient moxibustion is likely to result in a broader effective action range, deeper tissue penetration, and stronger therapeutic stimulation compared with moxa box moxibustion.

Acupoints serve as the loci for the ingress, egress, and accumulation of meridian qi and blood. Stimulation of these acupoints can facilitate the unblocking of meridians and modulate visceral functions (32). In this study, we selected CV4, BL23, BL20, BL21, BL31, BL32, BL33, and BL34 as the intervention acupoints. Specifically, moxibustion at CV4 is employed to warm the middle jiao and address deficiency (33), whereas BL23 is utilized to tonify kidney qi and alleviate consumptive fatigue (34). In alignment with Zhang Zhongjing’s recommendation to treat consumptive diseases primarily by regulating the spleen and stomach (35), BL20 and BL21 are applied to activate the middle jiao, enhance spleen–stomach qi, and replenish primordial qi (36, 37). In addition, moxibustion on BL31–BL34 is intended to nourish kidney yang and kidney essence (38). Consequently, the combined moxibustion at these acupoints is anticipated to yield yang-warming and deficiency-tonifying effects, thereby mitigating symptoms of CFS.

Fatigue is the primary symptom associated with CFS; therefore, the FS-14 will be used to assess the potential of the intervention in reducing the severity of fatigue experienced by patients. Beyond persistent fatigue, individuals with spleen–kidney yang deficiency often exhibit distinct TCM clinical manifestations, including aversion to cold, lumbosacral soreness and weakness, lassitude, and reduced food intake. Therefore, the Spleen–Kidney Yang Deficiency Symptom Scale will be employed to quantitatively evaluate the overall improvement in clinical symptoms associated with this TCM syndrome. Additionally, anxiety and depression are prevalent among patients with CFS, potentially exacerbating physical symptoms and negatively impacting clinical outcomes (39, 40). As such, the SDS and SAS will be used to assess the intervention’s effect on these negative emotional states.

Furthermore, this study aims to analyze the variations in the infrared thermal imaging characteristics of the Sanjiao and Governor Vessel before and after treatment. The Sanjiao plays a crucial role in the production, transformation, and distribution of qi, blood, body fluids, and essence, facilitating their upward, downward, outward, and inward movement. Through the transformation of qi by the Sanjiao, the zang-fu organs are interconnected, thereby regulating bodily functions and supporting all vital activities of the human body (41). In healthy individuals, the Sanjiao maintains a stable temperature rhythm, typically exhibiting an orderly gradient with a cooler upper jiao, a moderate middle jiao, and a warmer lower jiao, thereby preserving the physiological equilibrium of a cool upper and warm lower jiao (42). The Governor Vessel, often referred to as the “sea of all yang meridians,” plays a crucial role in regulating yang-qi throughout the body. It traverses the posterior midline and establishes a connection with the kidneys, thereby accumulating yang-qi to nourish internal organs and serving as a primary conduit for the transport of essence (43). Consequently, the surface infrared temperature and continuous thermal profile of the Governor Vessel can serve as indicators of whether systemic yang-qi is both sufficient and unobstructed (19, 44). Patients suffering from CFS typically exhibit distinct features in infrared thermal imaging, such as significant thermal deviations in the upper jiao, cooler deviations in the middle jiao, and a lack of continuity in the thermal line of the Governor Vessel (45). Therefore, detecting the infrared thermal characteristics of the Sanjiao and Governor Vessel can provide an objective assessment of the regulatory effects of moxibustion on yang-qi distribution and meridian thermal balance, while also offering insights into its underlying mechanism in the treatment of CFS.

Nevertheless, this trial is subject to several limitations. First, due to the distinct differences in operational methodologies and appearance between the Jianqiao ancient moxibustion and moxa box moxibustion interventions, it is not feasible to achieve complete blinding of therapists and participants. This limitation may introduce performance bias. To mitigate such bias, all outcome evaluators and statisticians will remain blinded throughout the trial process. Second, the study is a single-center trial with a relatively small sample size, which may limit the generalizability of the findings to broader populations and different clinical settings. Future research should consider multicenter trials with larger sample sizes to enhance validation. Third, this trial will exclusively recruit patients with CFS exhibiting a spleen–kidney yang deficiency pattern. Other prevalent TCM patterns, such as liver stagnation and spleen deficiency, heart-spleen deficiency, and internal dampness-phlegm retention, are not addressed. Consequently, the results cannot be generalized to CFS patients with other syndrome types.
